# The burdock database: a multi-omic database for *Arctium lappa*, a food and medicinal plant

**DOI:** 10.1186/s12870-023-04092-3

**Published:** 2023-02-09

**Authors:** Yueyue Song, Yanyun Yang, Liang Xu, Che Bian, Yanping Xing, Hefei Xue, Wenjuan Hou, Wenxiao Men, Deqiang Dou, Tingguo Kang

**Affiliations:** School of Pharmacy, Liaoning University of Traditional Chinese Medicine, Dalian, 116600 China

**Keywords:** *Arctium lappa*, Database, Genome, Transcriptome, Tools

## Abstract

**Background:**

Burdock is a biennial herb of Asteraceae found in Northern Europe, Eurasia, Siberia, and China. Its mature dry fruits, called *Niu Bang Zi*, are recorded in various traditional Chinese medicine books. With the development of sequencing technology, the mitochondrial, chloroplast, and nuclear genomes, transcriptome, and sequence-related amplified polymorphism (SRAP) fingerprints of burdock have all been reported. To make better use of this data for further research and analysis, a burdock database was constructed.

**Results:**

This burdock multi-omics database contains two burdock genome datasets, two transcriptome datasets, eight burdock chloroplast genomes, one burdock mitochondrial genome, one *A. tomentosum* chloroplast genome, one *A. tomentosum* mitochondrial genome, 26 phenotypes of burdock varieties, burdock rhizosphere-associated microorganisms, and chemical constituents of burdock fruit, pericarp, and kernel at different growth stages (using UPLC-Q-TOF–MS). The wild and cultivation distribution of burdock in China was summarized, and the main active components and pharmacological effects of burdock currently reported were concluded. The database contains ten central functional modules: Home, Genome, Transcriptome, Jbrowse, Search, Tools, SRAP fingerprints, Associated microorganisms, Chemical, and Publications. Among these, the “Tools” module can be used to perform sequence homology alignment (Blast), multiple sequence alignment analysis (Muscle), homologous protein prediction (Genewise), primer design (Primer), large-scale genome analysis (Lastz), and GO and KEGG enrichment analyses (GO Enrichment and KEGG Enrichment).

**Conclusions:**

The database URL is http://210.22.121.250:41352/. This burdock database integrates molecular and chemical data to provide a comprehensive information and analysis platform for interested researchers and can be of immense help to the cultivation, breeding, and molecular pharmacognosy research of burdock.

## Background

*Arctium lappa* L., a medicinal and edible plant of Asteraceae, is native to Northern Europe, Eurasia, Siberia, and China. Because of its rich nutritional value, it enjoys the reputation of being the king of vegetables in Europe. The burdock root is consumed as a vegetable in Europe and Japan, while its fruit has a long medicinal history in China. The dried, ripe fruit is piquant or bitter to the taste and has cold properties associated with the lungs and stomach meridians. It is traditionally used to disperse wind–heat, detoxify and purify blood, and treat common cold, cough, sore throat, measles, and rash [1, 2]. Burdock was first described in the *Mingyibielu* and is a commonly used traditional medicine since ancient times [3]. In *Taipingshenghuifang*,* Niu Bang Zi San* (burdock powder) is prescribed to treat febrile conditions and gastrointestinal disorders resulting from heat and poison attacks. Furthermore, Fructus Arctii is acrid and neutral in nature and serves as an adjunct medicine to *Yin Qiao San* (Lonicera and Forsythia powder), as stated in the *Wenbingtiaoli*. It moistens the lungs, disperses wind, clears heat, and relieves sore throat. Several chemical components are present in burdock, with lignans, volatile oils, fatty acids, terpenoids, and phenolic acids being the primary components [4]. Therefore, Fructus Arctii has several antitumor, anti-inflammatory, antidiabetic, antibacterial, and antiviral properties. It is clinically used to treat cold, cough, phlegm, measles, rubella, sore throat, and other diseases [5]. Many Chinese patent medicines are made of burdock using modern pharmaceutical technology, such as Yanshu capsule, Yanshu oral liquid, Qingre Qudu pill, vitamin C Yinqiao granules and so on [6, 7].

Asteraceae is one of the largest angiosperm families, including more than 1000 genera. It is widely distributed in the world, with most species having important economic value as ornamental, food, and medicinal plants. The family has the highest evolutionary status among dicotyledons [8]. Therefore, with the continuous development and improvement of sequencing technology in recent years, many Asteraceae plant genomes have been completely sequenced to study the evolution of biological groups. These include *Artemisia annua* [9], lettuce (*Lactuca sativa*) [10], sunflower (*Helianthus annuus*) [11], artichoke (*Cynara cardunculus*) [12], *Mikania micrantha* [13], *Carthamus tinctorius* [14], *Stevia rebaudiana* [15], chicory (*Cichorium intybus*), endive (*C. endivia*), great burdock (*A. lappa*), yacon (*Smallanthus sonchifolius*) [16], and *Erigeron breviscapus* [17]. Our laboratory also performed complete genome sequencing of burdock. Approximately 1.70 Gb (95.4%) of the contig sequences were anchored onto 18 chromosomes using Hi-C data [18].

Wei et al. (2020) sequenced the transcriptome of the burdock root, laying an experimental foundation for the identification of burdock functional genes, analysis of secondary metabolic pathways, and research on its regulatory mechanism [19]. Our laboratory performed transcriptome sequencing of 10 burdock samples (five growth stages of burdock fruits, leaves, stems, perianths, petioles, and roots) to study the biosynthetic pathway of arctiin and key enzyme genes in burdock [20]. Based on genome data, transcriptome analysis was also performed on the roots of burdock at different growth stages. By comparative transcriptome analysis, our research team preliminarily speculated on the regulatory mechanism of secondary metabolite biosynthesis of burdock and found three proteins highly related to the accumulation of arctiin: 4-coumarate-CoA ligase (4CL), dirigent protein (DIR), and hydroxycinnamoyl transferase (HCT) [18]. However, the synthetic pathway of arctiin has not been verified based on the current research results.

Accessing large and complex data efficiently and accurately to conduct research is a problem to be solved. At present, online databases include the Cucurbitaceae database (http://www.gourdbase.cn/) [21], Bayberry database (http://www.bayberrybase.cn/) [22] and Malvaceae database (http://magen.whu.edu.cn/) [23]. To further explore the biosynthetic pathway, breeding, and ecology-related research of burdock, we developed a burdock multi-omics database. For convenience, we compiled the phenotypic, genomic, transcriptomic, and chemical research data of burdock to develop a user-friendly burdock database. The database also contains tools for researchers, such as genome browser, sequence alignment, homologous protein prediction, and primer design.

## Utility and discussion

### Burdock database content

**Fig. 1:**
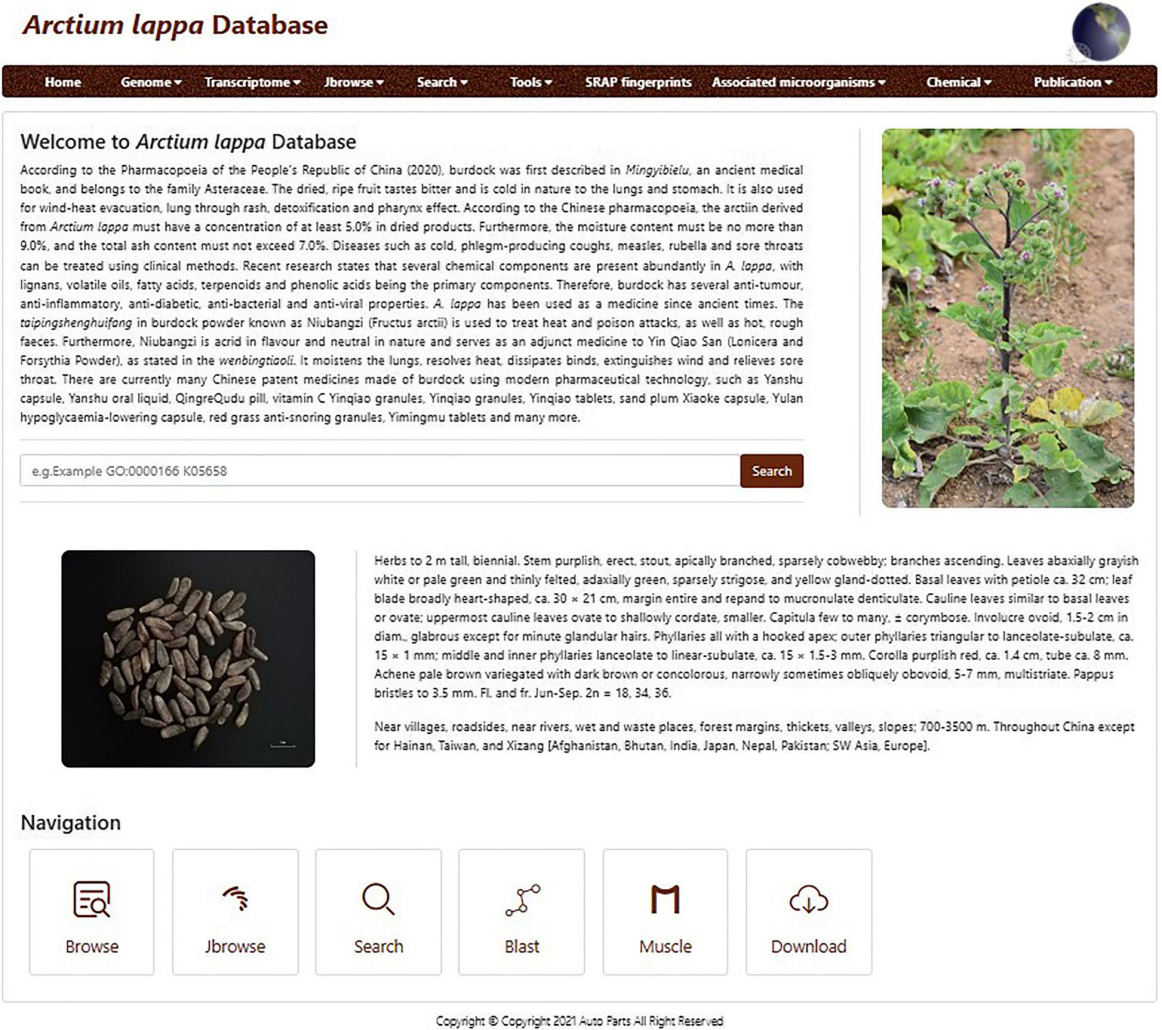
Burdock database homepage. Ten main modules displayed at the top of the interface include “Home”, “Genome”, “Transcriptome”, “Jbrowse”, “Search”, “Tools”, “SRAP fingerprints”, “Associated microorganisms”, “Chemical”, and “Publications”

**Fig. 2:**
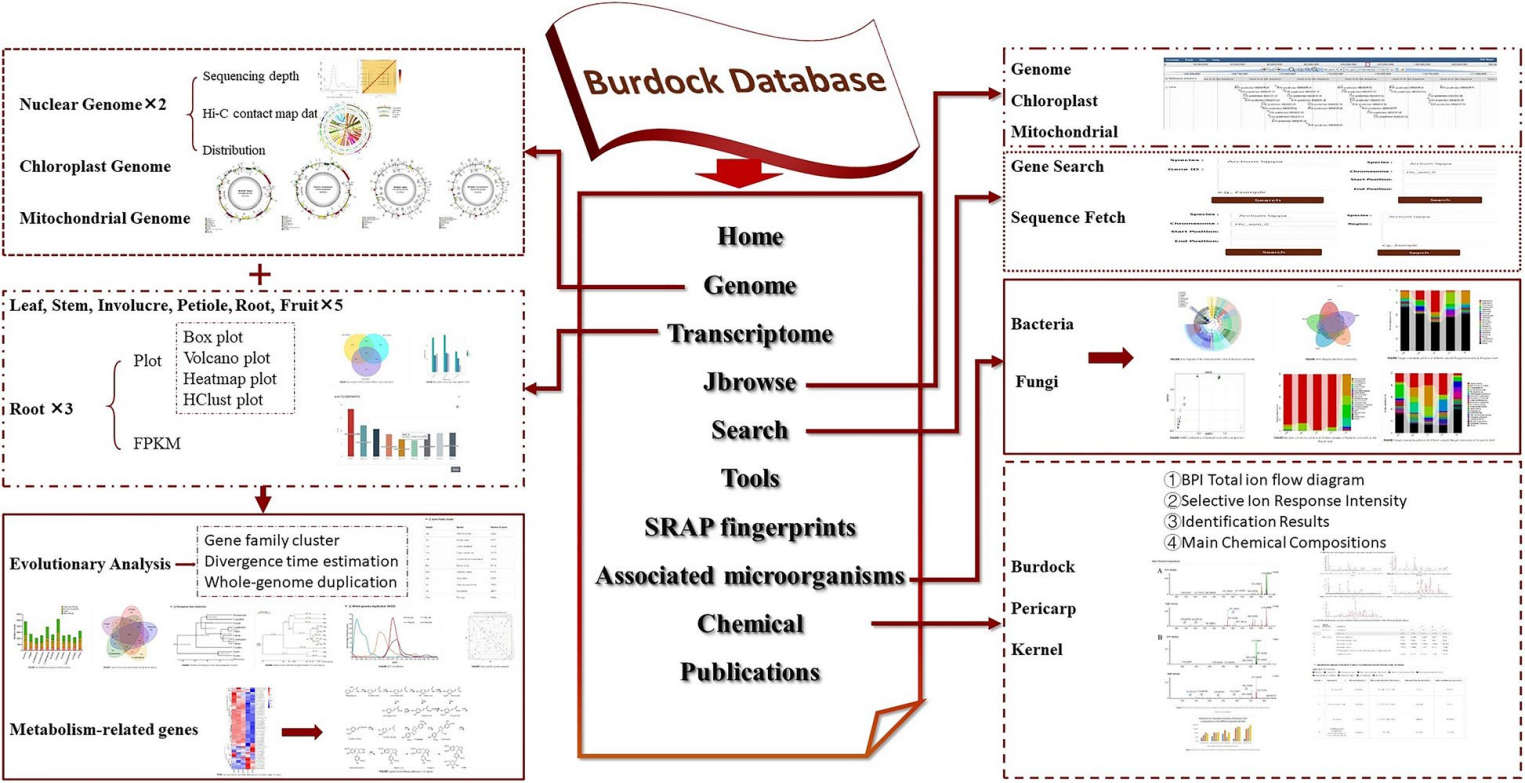
Schematic of the burdock database

The burdock database contains ten central functional modules: Home, Genome, Transcriptome, Jbrowse, Search, Tools, SRAP fingerprints, Associated microorganisms, Chemical, and Publications (Fig. 1). It includes two genome datasets, two transcriptome datasets, eight burdock chloroplast genomes, one burdock mitochondrial genome, one *A. tomentosum* chloroplast genome, one *A. tomentosum* mitochondrial genome, 26 phenotypes of burdock varieties [24–27], distribution of burdock resources in China, burdock rhizosphere-associated microorganisms [28], 18 pairs of sequence-related amplified polymorphism (SRAP) primers [27], and chemical constituents of burdock fruit, pericarp, and kernel at different growth stages (using UPLC-Q-TOF–MS) [20, 29]. In addition, the summary and conclusion on 9 reported pharmacological effects of burdock have been realized. In the “Publications” module, 120 relevant papers and a book, *The Study of Burdock in China*, can be downloaded for research purposes. The database also contains intuitive and convenient tools to facilitate data retrieval (Fig. 2).

### Applications of the burdock database

#### Genome

This module contains the first high-quality chromosome-level draft genome of *A. lappa* obtained using Illumina and PacBio sequencing data. The assembled genome is approximately 1.79 Gb, with 1.12 Gb (68.46%) of repetitive sequences, 32,771 protein-coding genes, and 616 positively selected candidate genes [18]. Another published burdock genome, with assembled the contigs of approximately 1.73 Gb, is also included [16]. This module includes the sequencing results of eight complete chloroplast genomes of burdock, with a full length between 152,767 bp and 163,851 bp, and 90 annotated protein-coding genes. It also includes the complete chloroplast genome of *A. tomentosum*, a close source plant of burdock, with a total length of 152,688 bp, and 89 annotated protein-coding genes [30]. A burdock chloroplast genome dataset from the NCBI database (accession number MH161419) is also present. The module includes one burdock and one *A. tomentosum* mitochondrial genome, with similar genome sizes of 312,598 bp and 312,609 bp, respectively, and 76 and 75 annotated protein-coding genes, respectively [31]. The module summarizes the phenotypic characteristics of 26 burdock cultivars reported thus far, and it contains the name, origin, root characteristics, leaf color, and maturity descriptions of these cultivars [24–27]. To better develop and utilize the medicinal resources of burdock, the module further summarized the main wild and cultivation conditions in China.

**Fig. 3:**
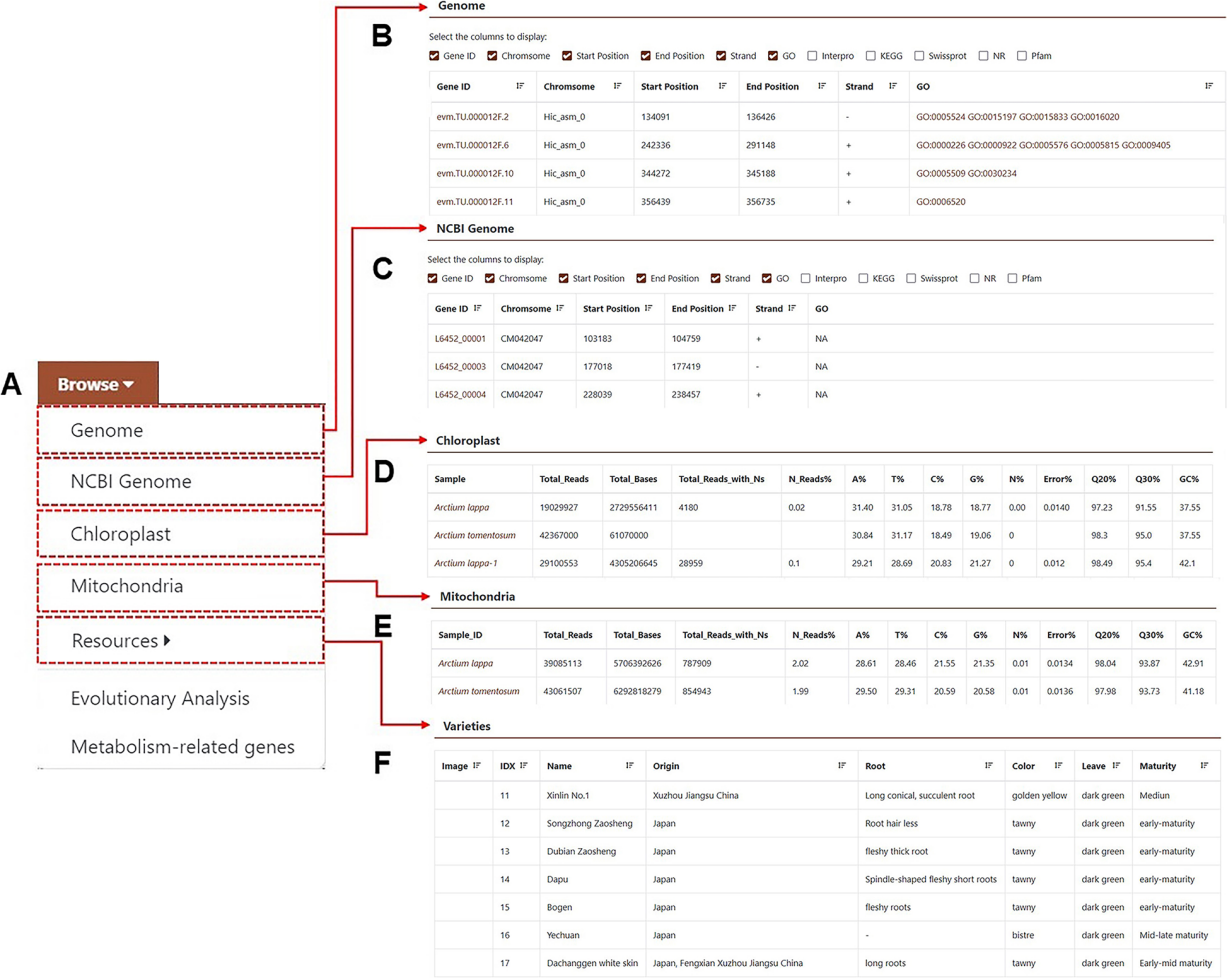
Details of the “Browse” module. **A** Sub-modules include “Genome”, “NCBI Genome”, “Chloroplast”, “Mitochondria”, “Varieties”, “Evolutionary Analysis”, and “Metabolism-related genes”. **B**-**F** Functions of “Genome”, “NCBI Genome”, “Chloroplast”, and “Varieties” are presented from top to bottom

To explore, users can click the “Genome” tab in the navigation bar, where “Genome”, “NCBI Genome”, “Chloroplast”, “Mitochondria”, “Resources”, “Evolutionary Analysis”, and “Metabolism-related genes” appear in the drop-down list (Fig. 3A). Users can click any label on this list according to their needs. After clicking “Genome”, the page shows the chromosome position, start and end positions of each gene, as well as the protein annotation and sequence number of genes in the GO, InterPro, KEGG, Swiss-Prot, NR, and Pfam databases (Fig. 3B). “NCBI Genome”, “Chloroplast”, and “Mitochondria” are similar (Fig. 3C-F). With this module, users can learn the basics of each gene in the burdock genome in detail. The gene ID is clickable and links to the “Jbrowse” module for visualising gene information. Moreover, a click on the ID number of each protein database will link to the corresponding database. “Evolutionary Analysis” shows the results of phylogenetic estimation of the divergence time of protein-coding genes in 11 sequenced genomes (including burdock), and the expansion and contraction results of 11 plant gene families. “Metabolism-related genes” shows the predicted biosynthetic pathways of lignan and lignin in burdock based on current research results. To verify this prediction, the module collected 18 kinds of enzymes related to lignans and lignin biosynthesis pathway recently reported. Through comparing, the module will have those with corresponding domains as candidate genes. The database showed the results of heat map analysis of candidate genes at five different growth stages of the burdock fruit. With the corresponding gene ID indicated in the figure, users can search for such an ID and then download the sequence if they want to perform qPCR validation for the results. All the gene sequences in this module can be downloaded, which can be used for analysis of individual or population variation information of burdock, such as single nucleotide polymorphism (SNP), insertion deletion (InDel) and copynumber variation (CNV); Obtain molecular genetic characteristics, further predict candidate genes for economic traits, and analyze genetic evolution.

#### Transcriptome

**Fig. 4:**
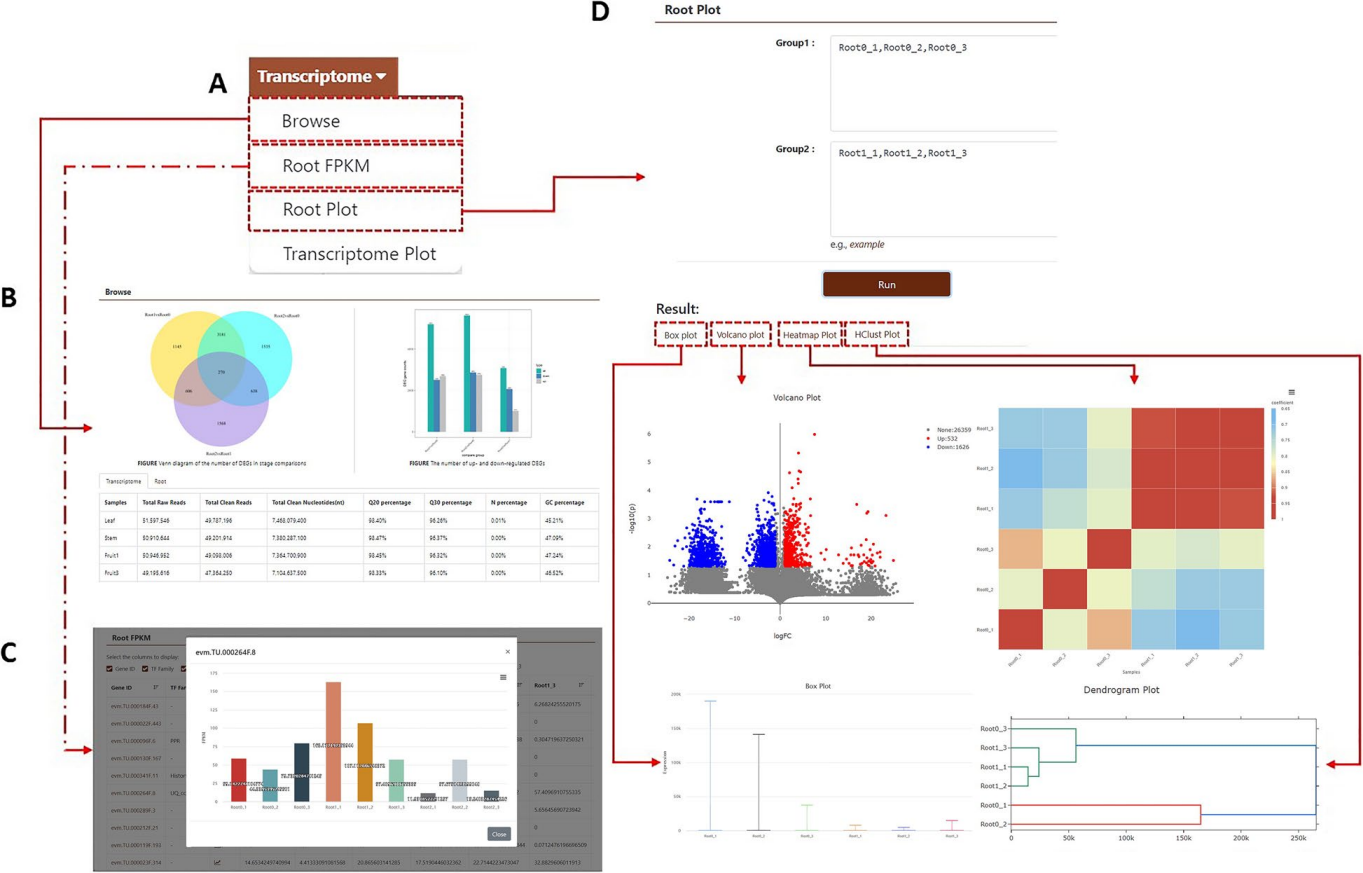
Detailed description of the “Transcriptome” module.** A** Sub-modules include “Browse”, “Root FPKM”, “Root Plot”, and “Transcriptome Plot”. **B** “Browse” for content. **C** Expression level of 32,771 genes in roots at different developmental stages (seedling, annual, and biennial). **D** With “Root0 versus Root1” as an example, the content of “Root Plot” is displayed

This module contains RNA-seq datasets from different tissues of burdock at different growth stages, including fruit (bud, early flowering, full flowering, late flowering, mature), perianth, stem, petiole, involucre, leaf, root (seedling, annual, and biennial), and stalk (Fig. 4). The database presents the results of comparative transcriptome analysis and candidate differentially expressed genes (DEGs) for roots at different developmental stages as a bar chart (Fig. 4C) [18]. Gene ID under this module can also be directly linkable to the “Jbrowse” module and can be downloaded. The database also supports four visual analysis charts (“Box plot”, “Volcano plot”, “Heatmap plot”, and “HClust plot”) that show the gene expression levels of fragments per kilobase of transcript per million fragments mapped (FPKM) in the different tissues described above. Users must click the “Transcriptome” tab in the navigation bar and select “Root Plot” or “Transcriptome Plot” in the drop-down list. Taking “Root0 versus Root1” as an example, users can click the different options in the horizontal results bar to display the comparison results of the four charts (Fig. 4D). Users can visually identify the differences in expression at different sites.

#### Jbrowse

**Fig. 5:**
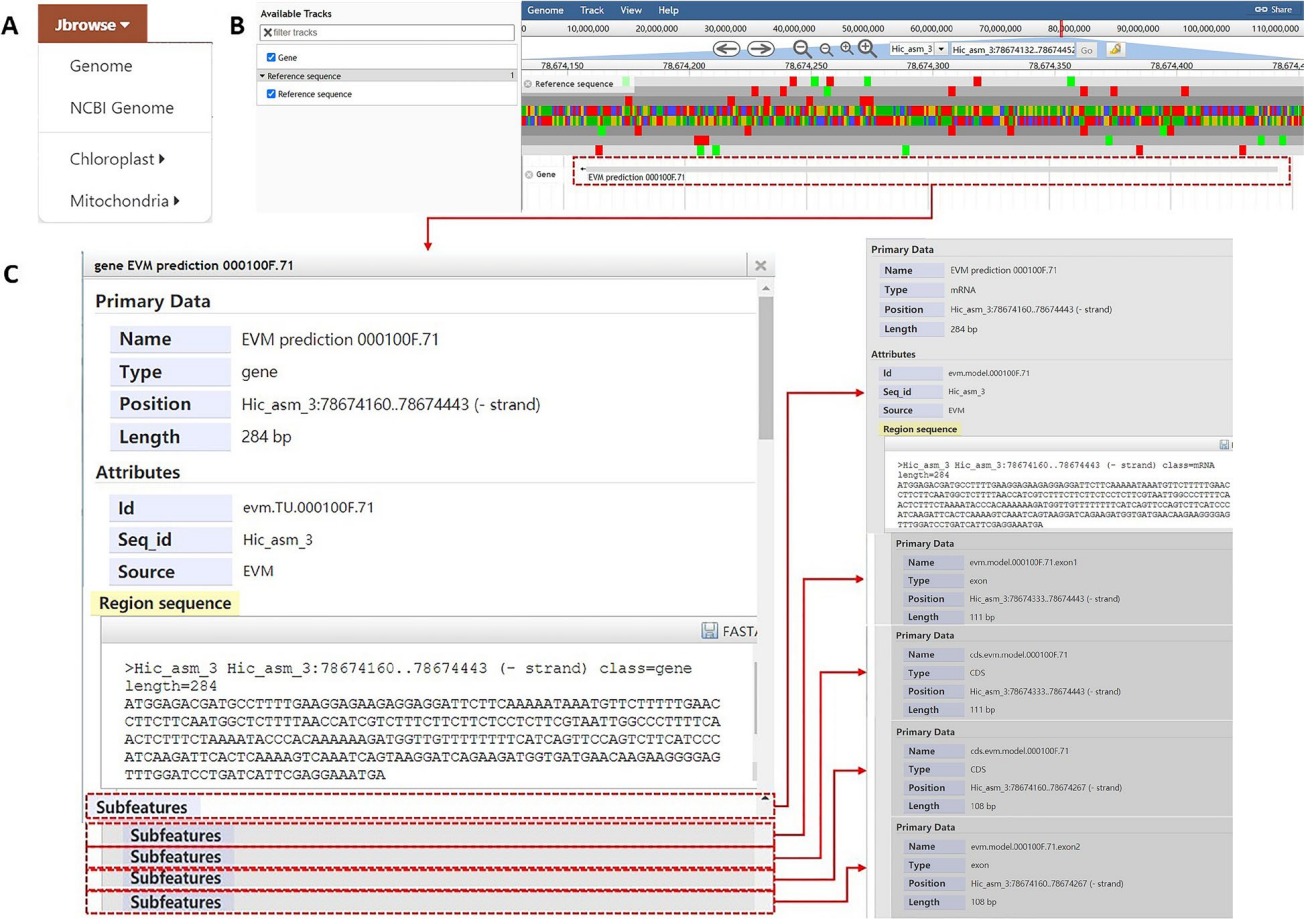
Detailed description of the “Jbrowse” module. **A** Sub-modules include “Genome”, “NCBI Genome”, “Chloroplast”, and “Mitochondria”. **B** The “Genome” interface. **C** Example of “EVM prediction 000100F.71”

The “Jbrowse” module of the database uses JBrowse, a genome visualization tool, to display two nuclear genomes of burdock and the chloroplast and mitochondrial genomes of burdock and its near source species *A. tomentosum* (Fig. 5A). Users can select a specific region of the desired chromosome in the search box above, and click the “Go” button to browse. For example, Fig. 5B shows the 78,674,134–78,674,445 bp interval on chromosome “Hic_asm_3” (Fig. 5B). As shown in Fig. 5C, users can click the “EVM prediction 000100F.71” gene, and the transcript of the gene will be displayed on the page, showing the transcript information of all exons and introns of the gene. A FASTA format file can also be downloaded (Fig. 5C). Clicking on gene IDs obtained from other modules can also link users to this tool.

#### Search

**Fig. 6:**
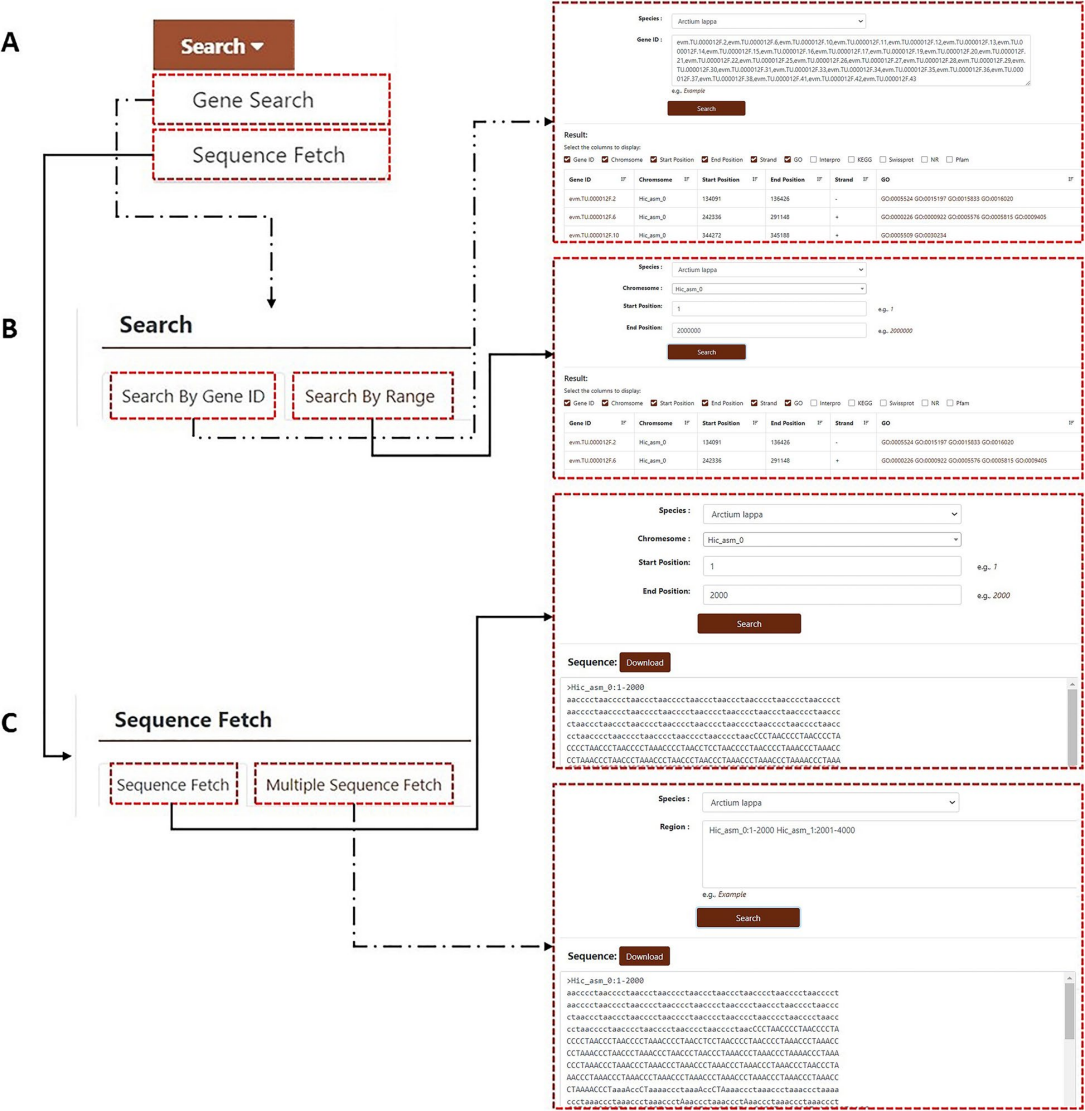
Detailed description of the “Search” module. **A** Sub-modules include “Gene Search” and “Sequence Fetch”. **B** Example of “Gene Search”. **C** Example of “Sequence Fetch”

The burdock database contains two genomes of size 1.79 [18] and 1.73 Gb [16]. The search module contains two sub-pages: gene search and sequence acquisition (Fig. 6A). On the gene search page, users can search by gene ID or range. The search results in the following information: “Gene ID”, “Chromosome”, “Start Position”, “End Position”, “Strand” and the annotation of genes in six protein databases: “GO”, “InterPro”, “KEGG”, “Swiss-Prot”, “NR”, and “Pfam” (Fig. 6B). Users can click “Sequence Fetch” to download the nucleotide sequence in the specified region. They can enter the start and end points of the sequence into the search box, and click “Download” to download all sequences in that range. To download multiple sequences, click “Multiple Sequence Fetch” in the secondary navigation to enter all sequences into the “Region” search box, and multiple sequences can be downloaded simultaneously (Fig. 6C).

#### Tools

**Fig. 7:**
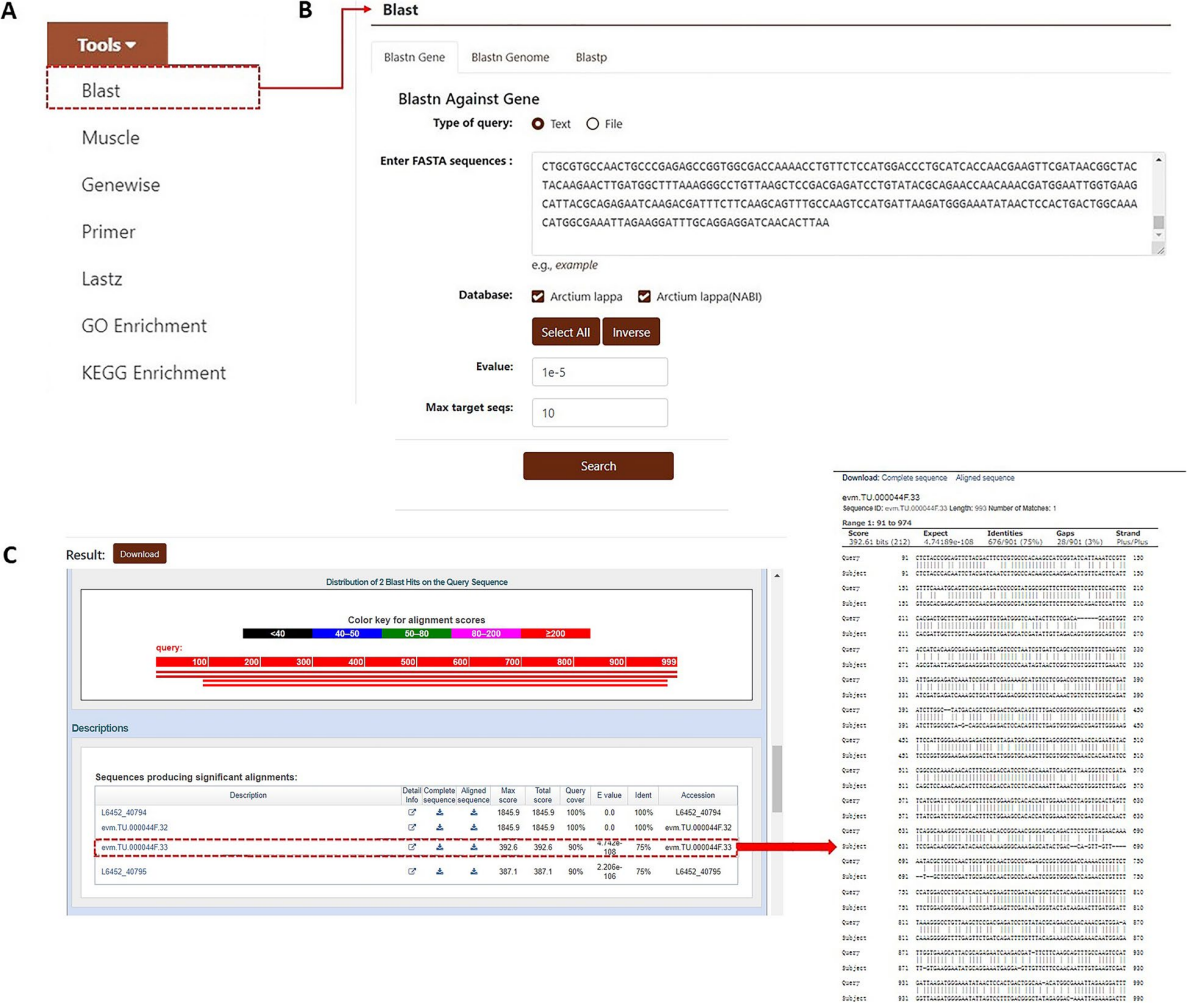
Introduction to the “Blast” tool in the “Tools” module. **A** Sub-modules include “Blast”, “Muscle”, “Genewise”, “Primer”, “Lastz”, “GO Enrichment”, and “KEGG Enrichment”. **B** Nucleotide sequences in the database compared using “Blastn”. **C** Results of nucleotide sequence alignment

The burdock database also has applications including “Blast”, “Muscle”, “Genewise”, “Primer”, “Lastz”, “GO Enrichment”, and “KEGG Enrichment” to meet most data processing requirements (Fig. 7A). The “Blast” module can perform homology alignment with the two burdock genomes in the database. The two kinds of alignment tools “Blastn” and “Blastp” can be used to perform nucleotide and protein sequence alignment. Users can click on “Blast”, and select “Blastn Gene”, “Blastn Genome”, and “Blastp” in the secondary navigation as required. They can enter the nucleotide or protein sequence in the FASTA format into the search box and click the “Search” button below to obtain the alignment results and download the sequence as required (Fig. 7). Users can obtain possible gene sequences by using this tool for comparison and screening if they want to research a certain type of burdock gene.

**Fig. 8:**
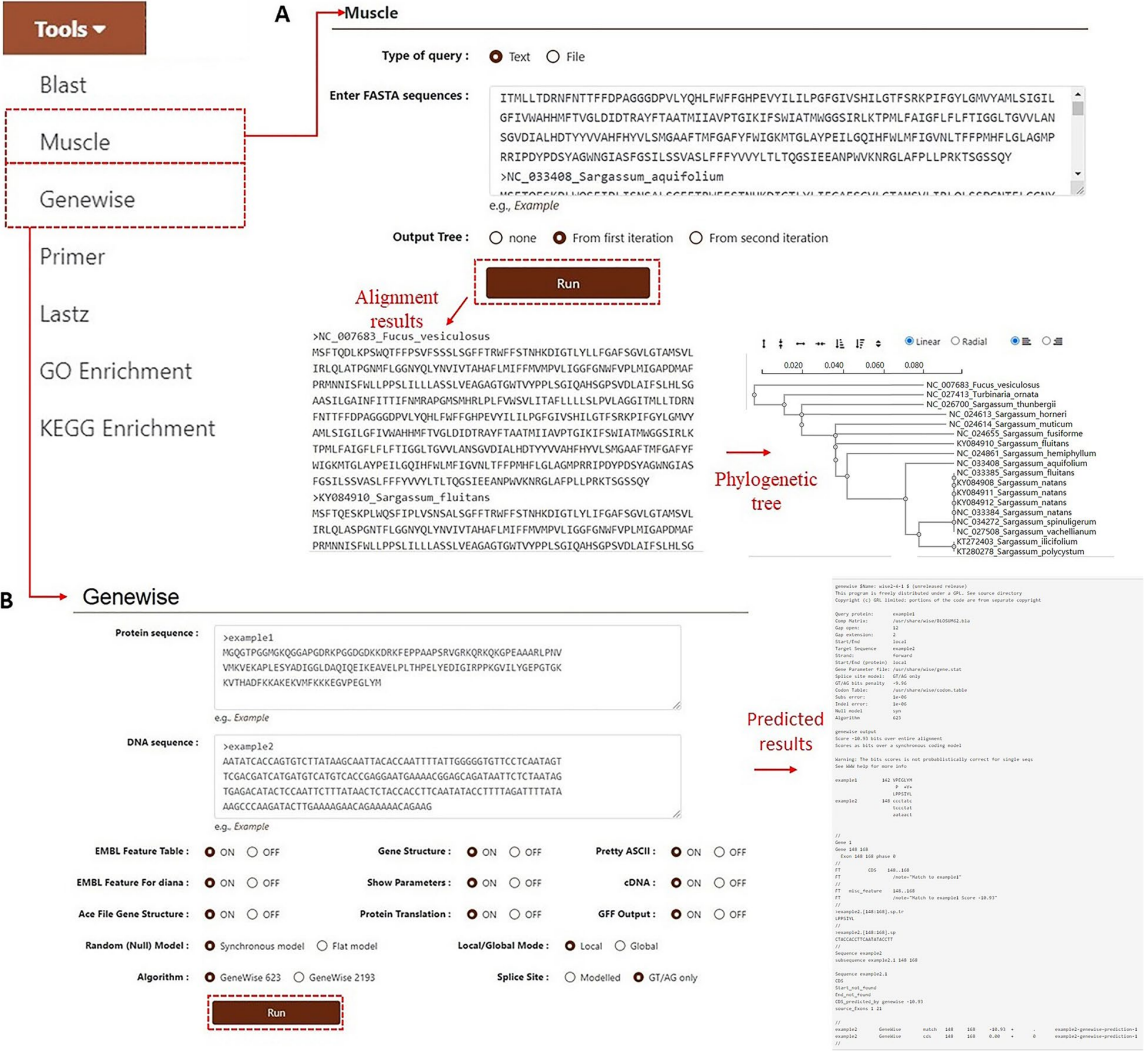
**A.** “Muscle” displaying alignment results and the phylogenetic tree. **B.** “Genewise” showing the prediction of homologous proteins

Users can click on “Muscle” to perform multiple sequence alignment analysis of nucleotide or protein sequences. The database also supports the direct construction of the phylogenetic tree of alignment results using the maximum likelihood method (Fig. 8A). Users are not limited to burdock-related sequences included in the database but can also paste the sequences of other species into the search bar according to their experimental needs and then obtain the tree diagram results by following the above steps. For homologous protein prediction, users must click the “Genewise” option, enter the nucleotide and protein sequences in the FASTA format into the two search boxes, respectively, and click the “Run” button to obtain the results (Fig. 8B). This tool is also not limited to sequences in the database.

**Fig. 9:**
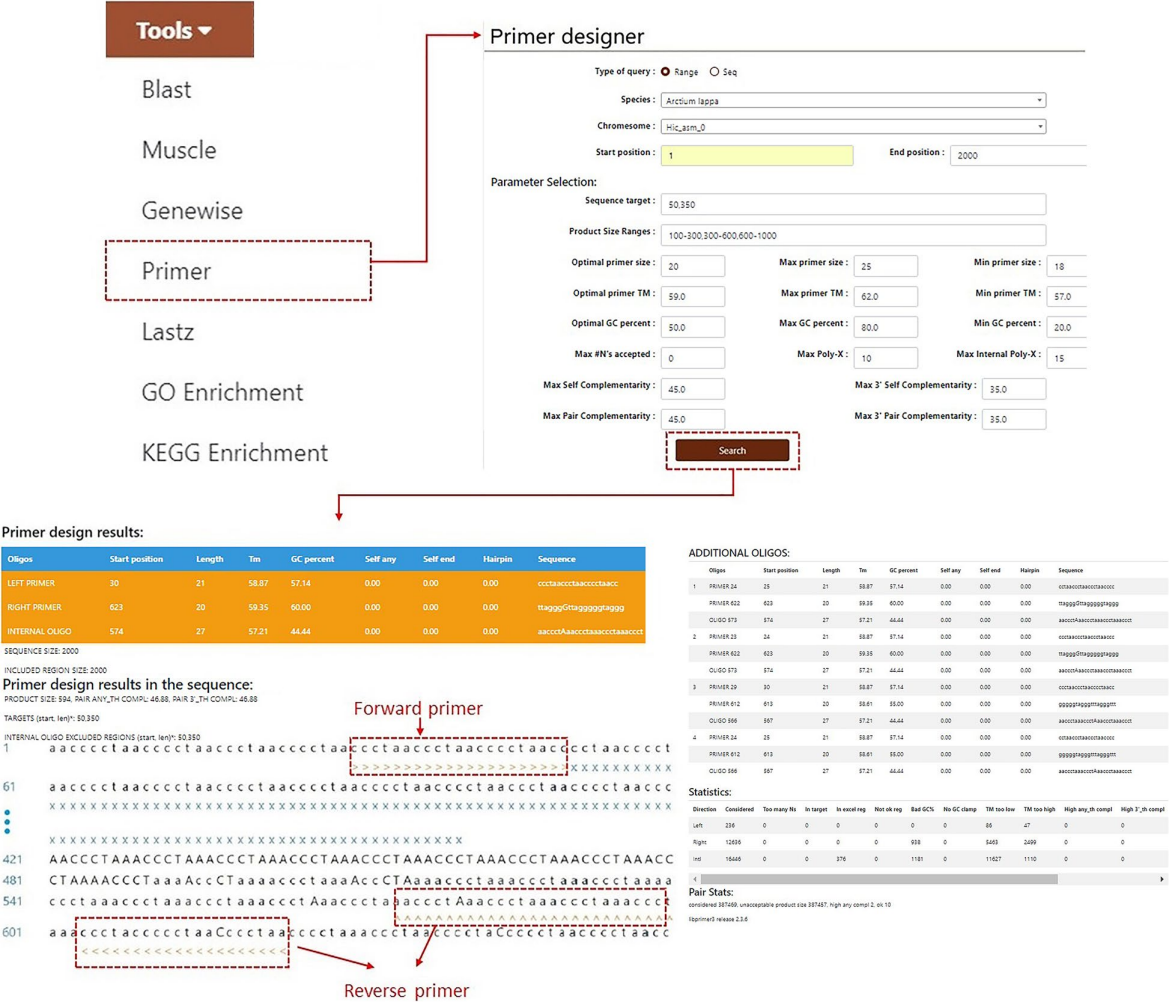
Example of the use of the “Primer” tool

If users require to amplify the burdock sequence, they can directly use the “Primer” function in the burdock database to design primers. Users can fill in the scope of the amplified sequence and the length and other requirements of the primer in the search box, and click the “Search” button (Fig. 9). If other species sequences require to be amplified, this function can also be used to design primers. Users can select the “Seq” radio button on the first line “Type of query”, and a FASTA search box will appear below. They can then enter the sequence, fill in the primer length and other requirements, and click the “Search” button. Users can only use this database to complete the search of the burdock target sequence, and then directly complete the comparison and primer design.

**Fig. 10:**
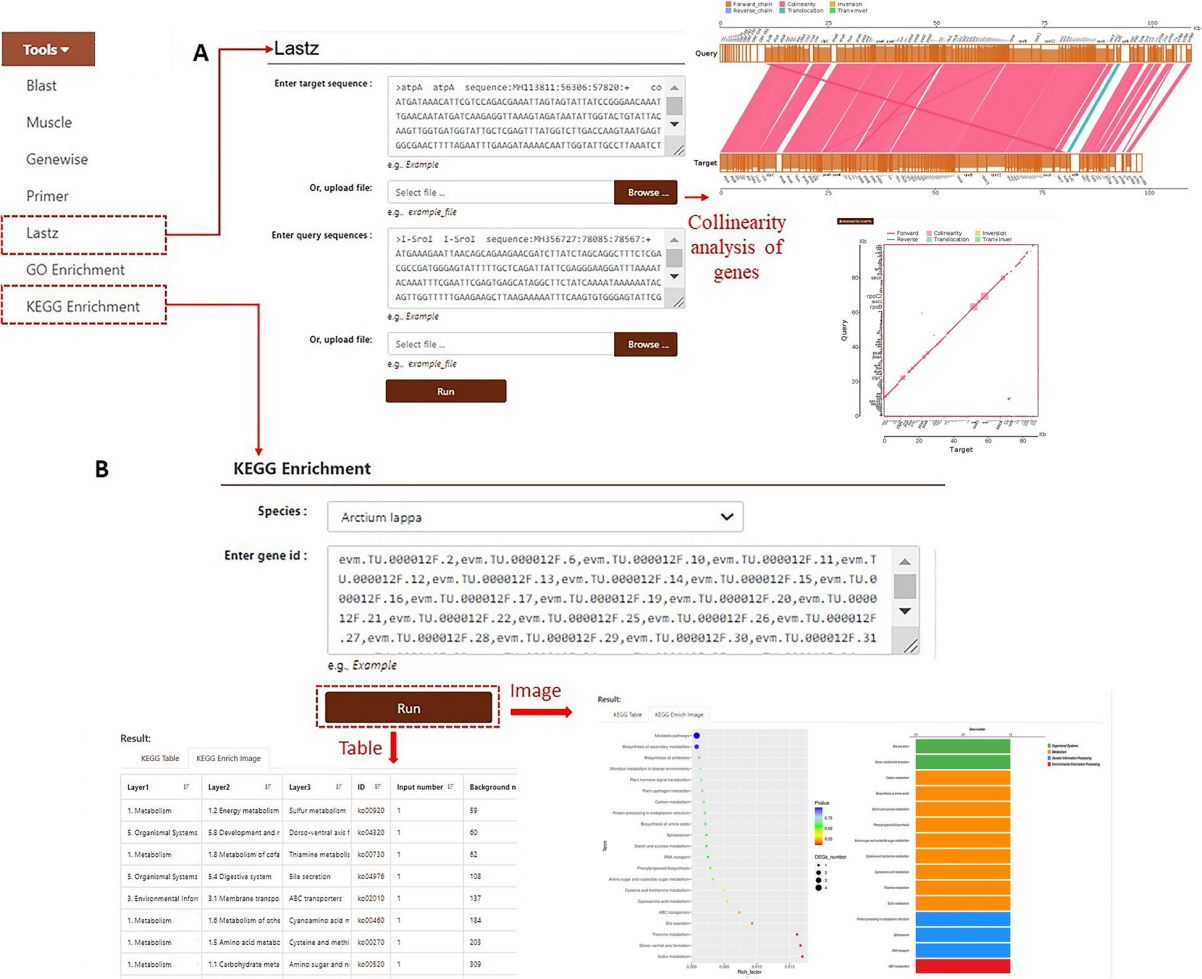
**A** “Lastz” displaying the results of gene collinearity analysis. **B** Example of the use of “KEGG Enrichment”

The burdock database can also meet the requirements of large-scale genome analysis. Users can click on “Lastz” in the navigation bar and enter the gene sequence in the FASTA format into the two search boxes. They can then click the “Run” button to obtain the results of the gene collinearity analysis (Fig. 10A). This module can fulfill the comparison task of users by using target sequences of other species. To study the biosynthetic pathways of secondary metabolites, the database also provides GO/KEGG enrichment analysis tools [27–29]. Users can enter the filtered DEGs into the search box and click the “Run” button. The locations of these annotated genes in the GO/KEGG database are presented in both tabular and pictorial formats (Fig. 10B). Users can enter gene IDs obtained from other modules to get their analysis results in two protein databases. It can be used for the functional study of genes and the screening of key genes.

#### SRAP fingerprints, Associated microorganisms, Chemical, and Publications

In the “SRAP fingerprints” module, 18 pairs of primers with high polymorphism and clear bands were screened by Geng et al*.* (2019) for burdock varieties [32]. SRAP marker technology is widely used in many kinds of vegetables and horticultural species. Users can use this result to easily identify burdock germplasm, which facilitates the breeding of burdock. The “Associated microorganisms” module contains microbial information on burdock root, stem, leaf, fruit, and rhizosphere soil. By high-throughput sequencing, 796,891 16S rRNA and 626,270 internal transcribed spacer reads were obtained from the samples. Using these reads, 922 bacterial species and 334 fungal species were detected. This page shows the distribution of bacteria and fungi in different tissues [33]. Users can use this data to study the formation mechanism of the secondary metabolites of burdock. The module of “Chemical” includes two submodules, “UPLC-Q-TOF–MS” and “Pharmaceutical Action”, of which, “UPLC-Q-TOF–MS” displays the identification results of chemical components in fruits, seeds, and pericarps of burdock by UPLC-Q-TOF–MS. From these, 31, 32, and 33 compounds were identified, and the content analysis of the key compounds in each part was included. This data can be used to understand the processes of burdock secondary metabolite accumulation [20, 34]. In the module of “Pharmacological Action”, the pharmacological actions of various chemical components of burdock in recent years were summarized. The “Publications” module of the database provides 120 research papers on burdock microscopic identification, chemical composition, herbal research, molecular identification, ecological suitability studies, cultivation, and varieties. *The Study of Burdock in China*, a book edited by Professor Tingguo Kang, is available for easy perusal.

## Conclusions

The burdock database is available at http://210.22.121.250:41352/. It aims to promote research of burdock in bioinformatics, molecular pharmacognosy, and genetic engineering. We hope to provide data support for research on the biosynthetic pathway of arctiin and other secondary metabolites as well as the genetic map of burdock. The multi-omics data in the database laid a foundation for the molecular breeding of burdock. It is convenient for experts and scholars to discover the key enzyme genes for the regulation and metabolism of drug efficacy and quality traits of burdock. It significantly improved the operability of polygenic traits improvement and the efficiency of new variety breeding. The establishment of the multi-omics database is of great significance to the understanding of the molecular mechanism of burdock growth and development and its position in the evolutionary process. With the development of bioscience and various technologies, the burdock database will be gradually enriched and provide more reference value for scholars. For now, you are welcome to browse and use the database for burdock research.

## Methods

The Burdock database was deployed in Ubuntu 16.04 operation system and developed by AKKA 2.6.5(webserver), MySQL 5.7(database server), Scala 2.13.2 and SBT 1.3.9. All data in database were managed and stored by MySQL Database Management System. The query function was enforced based on Slick 3.3.2 middleware tier. To visualize the genome, we used the Jbrowser 1.16.6. The website interface components were designed and implemented by the Bootstrap 4.6.0 and Play Framework 2.8.7. The website has been tested in several popular web browsers, including Firefox, Google Chrome and Internet Explorer.

## Data Availability

The database and web interface can be accessed at http://210.22.121.250:41352/.
